# Effects of diet type and supplementation of glucosamine, chondroitin, and MSM on body composition, functional status, and markers of health in women with knee osteoarthritis initiating a resistance-based exercise and weight loss program

**DOI:** 10.1186/1550-2783-8-8

**Published:** 2011-06-20

**Authors:** Teresa Magrans-Courtney, Colin Wilborn, Christopher Rasmussen, Maria Ferreira, Lori Greenwood, Bill Campbell, Chad M Kerksick, Erica Nassar, Rui Li, Mike Iosia, Matt Cooke, Kristin Dugan, Darryn Willoughby, LuAnn Soliah, Richard B Kreider

**Affiliations:** 1 Exercise & Sport Nutrition Lab, Department of Health & Kinesiology, Texas A&M University, College Station, TX 77843-4243, USA; 2 Human Performance Lab, Exercise & Sport Science Department, University of Mary-Hardin Baylor, Belton, Texas 76513, USA; 3 Department of Health, Human Performance and Recreation, Baylor University, One Bear Place, Box 97313, Waco, TX 76798-7313, USA; 4 Higuchi Biosciences Center, University of Kansas, Lawrence, KS 66047, USA; 5 School of Physical Education & Exercise Science, University of South Florida, Tampa, FL 33620, USA; 6 Department of Health and Exercise Science, University of Oklahoma, Norman OK 73019, USA; 7 Quality Improvement Programs, BlueCross and BlueShield of Texas, Dallas, TX 75266, USA; 8 Bouve College of Health, Northeastern University, Boston, MA 02115, USA; 9 Department of Health, Exercise Science, and Secondary Education, Lee University, Cleveland, TN 37320 m USA; 10 Schools of Biomedical & Health Sciences, Victoria University, Victoria University, Melbourne Victoria 8001, Australia; 11 Department of Family & Consumer Sciences, Baylor University, One Bear Place, Box 97346, Waco, TX 76798-73346, USA

## Abstract

**Background:**

The purpose of this study was to determine whether sedentary obese women with knee OA initiating an exercise and weight loss program may experience more beneficial changes in body composition, functional capacity, and/or markers of health following a higher protein diet compared to a higher carbohydrate diet with or without GCM supplementation.

**Methods:**

Thirty sedentary women (54 ± 9 yrs, 163 ± 6 cm, 88.6 ± 13 kg, 46.1 ± 3% fat, 33.3 ± 5 kg/m^2^) with clinically diagnosed knee OA participated in a 14-week exercise and weight loss program. Participants followed an isoenergenic low fat higher carbohydrate (HC) or higher protein (HP) diet while participating in a supervised 30-minute circuit resistance-training program three times per week for 14-weeks. In a randomized and double blind manner, participants ingested supplements containing 1,500 mg/d of glucosamine (*as d-glucosamine HCL*), 1,200 mg/d of chondroitin sulfate (*from chondroitin sulfate sodium*), and 900 mg/d of methylsulfonylmethane or a placebo. At 0, 10, and 14-weeks, participants completed a battery of assessments. Data were analyzed by MANOVA with repeated measures.

**Results:**

Participants in both groups experienced significant reductions in body mass (-2.4 ± 3%), fat mass (-6.0 ± 6%), and body fat (-3.5 ± 4%) with no significant changes in fat free mass or resting energy expenditure. Perception of knee pain (-49 ± 39%) and knee stiffness (-42 ± 37%) was decreased while maximal strength (12%), muscular endurance (20%), balance indices (7% to 20%), lipid levels (-8% to -12%), homeostasis model assessment for estimating insulin resistance (-17%), leptin (-30%), and measures of physical functioning (59%), vitality (120%), and social function (66%) were improved in both groups with no differences among groups. Functional aerobic capacity was increased to a greater degree for those in the HP and GCM groups while there were some trends suggesting that supplementation affected perceptions of knee pain (p < 0.08).

**Conclusions:**

Circuit style resistance-training and weight loss improved functional capacity in women with knee OA. The type of diet and dietary supplementation of GCM provided marginal additive benefits.

**Trial Registration:**

ClinicalTrials.gov: NCT01271218

## Background

According to the Centers for Disease Control and Prevention (CDC), there are approximately 43 million Americans suffering from arthritis with 21 million affected by osteoarthritis (OA) [1,2]. It is believed that 1 in 10 or 4.3 million adults aged 60 and older in the United States of America have symptomatic knee OA [3] and 1 in 4 individuals may develop knee and/or hip OA during their lifetime [2]. The general incidence and prevalence of OA increases two to tenfold from age 30 to 65 years [4]. By 2020, the CDC estimates that 60 million Americans will have OA [1,2]. Former athletes and active individuals have been reported to be susceptible to knee OA as they age [5].

Symptoms of OA include disability of the joints caused by swelling, pain after exercise or use, and joint stiffness [1,2]. Although the cause of OA is unknown, it is believed that stress placed upon the joints is a factor. Treatments for OA vary and have included rest, heat, anti-inflammatory and pain-relieving medications, corticosteroid injections, and/or surgery [5]. Physical activity has been suggested to be beneficial for OA patients while inactivity can serve as a risk factor for developing OA [5]. Research from the Framingham Knee Osteoarthritis Study indicated that overweight men and women have a higher risk for developing OA than those who are not overweight [6]. These researchers also reported that weight loss helped decrease pain associated with OA [7]. Messier and colleagues [8] reported that weight loss significantly reduces load exertion on the knee. Moreover, Miller and associates [9] reported that an intensive energy deficit diet combined with exercise training improved physical function indices in older obese adults with knee OA. It has been reported that changes in OA symptoms were best predicted by changes body fat [10]. In addition, reductions in strength relative to body weight can promote the development of OA [11]. As a result, interventions that strengthen the muscles and reduce body fat have been suggested to reduce pain and enhance functional capacity in individuals with OA [10,12,13].

Higher protein diets have been reported to promote greater weight loss while preserving fat free mass and resting energy expenditure to a greater degree than higher carbohydrate diets [14-16]. In addition, higher protein diets have been reported to promote greater improvement in several markers of health particularly in populations at risk to cardiovascular disease due to elevated glucose and/or triglyceride levels [17-19]. Prior research from our lab has indicated that 14-weeks of circuit style resistance-training while following a moderately hypo-energetic higher protein diet promoted significant reductions in weight and fat mass while improving fitness and markers of health in obese women [20,21]. A subsequent study indicated that this program was comparatively more effective in terms of promoting weight loss and improvements in markers of health and fitness than a meal replacement-based diet program with recommendations to increase physical activity [22]. Additionally, we have reported that higher protein diets promote more favorable changes in body composition and markers of health than a higher carbohydrate diet in obese women initiating training with and without insulin resistance [23]. Theoretically, adherence to a higher protein weight loss diet while participating in a resistance-training program may be more beneficial than a higher carbohydrate diet for patients with OA because it may promote greater reductions in fat mass, preserve fat free mass during weight loss, and promote greater improvements in functional status and markers of health.

Glucosamine sulfate supplementation in patients with knee pain has been reported to improve joint pain and function [24]. For example, Pavelka and colleagues [25] evaluated the effects of 3-years of glucosamine sulfate supplementation on progressive joint degeneration and symptoms associated with knee OA. Results indicated that markers of knee pain, physical function, and joints stiffness were improved. Similarly, Usha and coworkers [26] studied the efficacy and safety of combinations of glucosamine and methlysulfonylmethane (MSM) supplementation in patients with knee OA. The researchers found that supplementation with glucosamine and MSM reduced joint pain and swelling, while improving the physical function of the joints [26]. These findings and others indicate that glucosamine, chondroitin, and/or MSM supplementation may have some therapeutic benefits for OA patients. For this reason, dietary supplementation of glucosamine, chondroitin, and/or MSM has been recommended particularly for active individuals [5,27-29]. Theoretically, glucosamine, chondroitin, and MSM supplementation may provide additive benefits to individuals with knee OA initiating an exercise and weight loss program.

The purpose of this study was 1) to determine whether sedentary obese women with knee OA initiating an exercise and weight loss program will experience more favorable changes in body composition, functional status, and/or markers of health when following a higher protein diet compared to a higher carbohydrate-based diet; 2) to determine whether dietary supplementation of glucosamine, chondroitin, and MSM during a weight loss and exercise program lessens symptoms of pain, improves functional capacity, and/or promotes greater health benefits in women with knee OA; and, 3) to determine whether there are any additive benefits of combining these strategies. It was hypothesized that all participants would experience beneficial changes in body mass, body composition, functional status, and markers of health. However, greater benefits would be observed in those following a higher protein diet with glucosamine, chondroitin, and MSM supplementation.

## Methods

### Experimental design

The study was conducted as a randomized, double-blind, placebo-controlled parallel clinical trial conducted in a university research setting. Participants with physician diagnosed OA participated in the Curves^® ^(*Curves International, Waco, TX*) fitness and weight management program for 14-weeks [30]. This program was selected because it offers higher carbohydrate and higher protein diets; incorporates circuit-style resistance training as the primary exercise modality; it has been found to be effective in promoting weight loss and improving markers of health and fitness in sedentary obese women [20-23]; it offers a joint support supplement containing GCM to its members; and, the program is widely available. Participants were randomly assigned to ingest in a double-blind and randomized manner either a placebo or a commercially available dietary supplement containing glucosamine, chondroitin, and MSM. Primary outcome measures included measures of pain and functional capacity. Secondary outcome measures included weight loss and body composition; serum blood and hormones; and, measures of quality of life. All participants were tested for changes in energy intake; anthropometrics; body composition; resting energy expenditure; cardiovascular and muscular fitness; balance and functional capacity; serum and whole blood clinical markers; hormonal profiles; pain indices; and, psychosocial parameters after 0, 10, and 14 weeks of training, dieting, and supplementation.

### Participants

This research protocol was reviewed and approved by the university Institutional Review Board prior to initiation. Participants were recruited through area physicians, advertisements in local newspapers, campus flyers, and Internet advertisements. Interested participants were asked to contact the laboratory for an initial telephone pre-screening interview. General entrance criteria included being a female with physician diagnosed OA between the ages of 18-70 years with a body mass index (BMI) greater than 27 kg/m^2 ^and no recent participation in a diet or exercise program. Individuals who met initial entrance criteria were invited to attend a familiarization session in which the details of the study were explained, human subject consent forms were signed, and personal and medical history information obtained. Subjects were not allowed to participate in this study if they: 1.) were pregnant, became pregnant, or had a desire for pregnancy; 2.) had any metabolic disorder including known electrolyte abnormalities, heart disease, arrhythmias, diabetes, or thyroid disease; 3.) had a history of hypertension, hepatorenal, musculoskeletal, autoimmune, or neurological disease (other than knee OA); 4.) were taking thyroid, hyperlipidemic, hypoglycemic, or anti-hypertensive medications; 5.) had taken ergogenic levels of nutritional supplements that may affect muscle mass (e.g., creatine, HMB), anabolic/catabolic hormone levels (e.g., DHEA), or weight loss supplements (e.g., thermogenics) within three months prior to the start of the study; 6.) were ingesting any anti-inflammatory products two weeks before the start of the study or additional products during the study; 7.) reported any unusual adverse events associated with this study in which the supervising physician recommended removal from the study; 8.) had significant injury or surgery to the lower extremity or spine within the last six months; 9.) did not indicate a minimal amount of perceived pain and physical function limitation on inventories used in the study; 10.) had severe arthritis that required surgery and greatly limited functionality (inability to perform lunge); or, 11.) had arthritis that required the current use of physiotherapy modalities.

**Figure 1:**
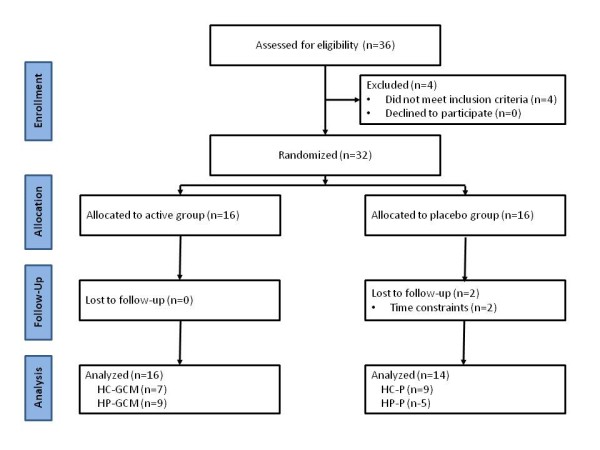
**Participant flow diagram**.

Figure 1 presents a flow diagram of study enrollment, allocation, follow-up, and analysis. A total of 42 women met initial phone screening criteria and were invited to familiarization sessions. Of these, 32 women met entrance criteria and were medially-cleared to participate in the study by a research nurse and their personal physician. A total of 30 women completed the study. Those who dropped out of the study did so due to time constraints unrelated to the exercise, diet, and/or supplementation program. Participants were 54 ± 9 years old, 163 ± 6 cm tall, weight 88.6 ± 13 kg, had a body fat percentage of 46.1 ± 3%, and had a BMI of 33.3 ± 5 kg/m^2^.

### Testing sequence

Participants underwent a detailed orientation and familiarization/practice session prior to baseline testing. This included an explanation of the methods of the study and how to adhere to the diet; an opportunity to practice testing procedures; and, familiarization to the exercise training equipment. Participants recorded all food and fluid intake on dietary record forms 4-days before each testing session for weeks 0, 10, 14. The dietary record included three days during the week and one weekend day. Participants were also asked to refrain from vigorous physical activity, alcohol intake, and ingestion of over the counter medications for 24-hours prior to testing. In addition, participants fasted for 12-hours prior to reporting to the laboratory. All testing was conducted in the early morning hours in order to control for diurnal variations in hormone levels.

Once reporting to the lab, participants completed a series of questionnaires that included the SF-36 quality of life (QOL) inventory; a Visual Analog Scale (VAS) to assess knee pain; and, the Western Ontario and McMasters University Osteoarthritis Index to assess knee function. Participants were then weighed, had total body water determined by multi-frequency bioelectrical impedance (BIA), and had body composition determined using dual-energy x-ray absorptiometry (DEXA). Following these assessments, participants had their blood pressure and resting heart rate determined using standard procedures. Participants then donated approximately 20 ml of fasting blood using venipuncture techniques of an antecubital vein in the forearm according to standard procedures. Following blood collection, participants had measurements taken of their knees to include knee circumference to determine swelling secondary to osteoarthritis and active range of motion to assess knee flexibility. The participants then performed sit to stand, step-up and over, and forward lunge balance and functional capacity assessments. Participants then performed a knee extension and flexion muscular strength and endurance test using an isokinetic dynamometer. Next, participants performed a maximal cardiopulmonary exercise stress test to assess symptom limited functional peak aerobic capacity. Finally, participants performed an upper body muscular strength and endurance bench press test. Participants completed weekly a medical safety/side effect report that was analyzed by the lab research nurse.

### Dietary intervention

All subjects followed the Curves^® ^exercise and weight loss program (*Curves International, Waco, TX*) that is designed to improve fitness and promote weight loss in women [30]. Participants were assigned to follow isoenergetic low fat diets with higher protein (HP) or higher carbohydrate (HC) macronutrient content based on their responses to a carbohydrate tolerance questionnaire as per diet guidelines. In both diets, participants were instructed to consume 1,200 kcals/d for 1-week (Phase I) and 1,600 kcals/d for 9-weeks (Phase II) during a 10-week active weight loss period. Participants following the HC diet were instructed to consume a diet containing 55% carbohydrate, 15% protein, and 30% fat. Subjects in the HP group were asked to follow a diet containing 7% carbohydrate, 63% protein, and 30% fat during Phase I of the diet and 15% carbohydrate, 55% protein, and 30% fat during Phase II of the diet. The final 4-weeks of the diet (Phase III) served as a weight maintenance period. Participants were instructed to consume 2,600 kcals d^-1 ^consisting of 55% carbohydrate, 15% protein, and 30% fat and to follow their respective Phase I diet (1,200 kcals/d) for 2-days only if they gained 1.35 kg (3 lbs) during the maintenance period. Participants were given diet plans and menus to follow at the start of the study and met with a registered dietitian and/or exercise physiologist at each testing session and every two weeks during the course of the study to discuss diet and exercise compliance. Previous research has demonstrated that this 14-week program promoted a 3-5 kg weight loss while maintaining resting energy expenditure in sedentary obese women [20-23].

### Supplementation protocol

Participants were randomly assigned to ingest in a double-blind manner caplets containing a commercially available supplement containing GCM (*Curves Joint and Connective Support™, Curves International, Waco, TX*) or a similarly prepared dextrose containing placebo (P) for double blind administration. The GCM supplement provided a total of 1,500 mg/d of glucosamine (*from d-glucosamine HCL*), 1,200 mg/d of chondroitin sulfate (*from chondroitin sulfate sodium*), 120 mg/d of niacin, 120 mg/d of sodium, 45 mg/d of zinc, 900 mg/d of MSM, 300 mg/d of boswellia serrata extract, 180 mg/d of white willow bark extract, and 15 mg/d of rutin powder. Participants ingested three caplets in the morning and the remaining three caplets in the evening 30-min before a meal for 14-weeks. The supplements were prepared in caplet form and packaged in generic bottles for double blind administration by Nutra Manufacturing (*Greenville, SC*). The dextrose placebo was prepared with a similar base material and color coated in order to have a similar appearance and aroma as the GCM supplement. Supplementation compliance was monitored by having the participants return empty bottles of the supplement at the end of each testing phase. In addition, internal monitoring of supplementation compliance occurred with participants signing a compliance statement in a post-study questionnaire.

### Training protocol

All subjects participated in the Curves supervised exercise program three days per week throughout the fourteen week protocol (a total of 42 workouts). Each circuit-style workout consisted of 14 exercises (e.g. elbow flexion/extension, knee flexion/extension, shoulder press/lat pull, hip abductor/adductor, chest press/seated row, horizontal leg press, squat, abdominal crunch/back extension, pec deck, oblique, shoulder shrug/dip, hip extension, side bends and stepping). The exercise machines contained calibrated pneumatic resistance pistons that allowed for opposing muscle groups to be trained in a concentric-only fashion. Participants were informed of proper use of all equipment and were instructed to complete as many repetitions in a 30-s time period. In a continuous, interval fashion, participants performed floor-based callisthenic (e.g. running/skipping in place, arm circles, etc.) exercises on recovery pads for a 30-s time period after each resistance exercise in an effort to maintain a consistent exercise heart rate that corresponded to 60% to 80% of their heart maximum heart rate. All workouts were supervised by trained fitness instructors who assisted with proper exercise technique and maintenance of adequate exercise intensity. Participants were required to complete two rotations through all exercises which corresponded to exercising for approximately 28-min followed by a standardized whole-body stretching routine. Compliance to the exercise program was set a priori at a minimum of 70% compliance (30/42 exercise sessions).

## Procedures

### Diet assessment

Participants recorded all food and fluid intake for four days prior to each testing session. This included three weekdays and one weekend day. Dietary inventories were reviewed by a registered dietitian and subsequently analyzed for average energy and macronutrient intake using the ESHA Food Processor (*Version 8.6*) Nutritional Analysis software (*ESHA Research Inc., Salem, OR*).

### Body composition

Height and body mass were determined according to standard procedures using a calibrated electronic scale (*Cardinal Detecto Scale Model 8430, Webb City, Missouri*) with a precision of +/-0.02 kg. Intracellular, extracellular, and total body water was assessed using a Xitron 4200 Bioelectrical Impedance Analyzer (*Xitron Technologies, Inc., San Diego, CA*) in order to monitor hydration status among testing sessions. Bone density and body composition (excluding cranium) were assessed using a Hologic Discovery W (*Hologic Inc., Waltham, MA*) dual energy x-ray absorptiometer (DXA) equipped with APEX Software (*APEX Corporation Software, Pittsburg, PA*). Mean coefficients of variation for bone mineral content and bone mineral density measurements performed on the spine phantom ranged between 0.41 - 0.55%. Test-retest reliability studies performed with this DXA machine have previously yielded mean coefficients of variation for total bone mineral content and total fat free/soft tissue mass of 0.31 - 0.45% with a mean intra-class correlation of 0.985 [31].

### Resting energy expenditure

Resting energy expenditure (REE) was assessed using a ParvoMedics TrueMax 2400 Metabolic Measurement System (*ParvoMedics, Inc., Sandy, UT*). This test was a non-exertional test performed in a fasted state with the participants lying supine on an exam table. A clear, hard plastic hood and soft, clear plastic drape was placed over the participants' neck and head in order to determine resting oxygen uptake and energy expenditure. All participants remained motionless without falling asleep for approximately 20 minutes. Data were recorded after the first ten minutes of testing during a five minute period of time in which criterion variables (e.g., VO_2 _L/min) changed less than 5%. Test-retest measurements on 14 participants from a study previously reported [20] revealed that test-retest correlations (r) of collected VO_2 _in l/min ranged from 0.315 - 0.901  and coefficient of variation ranged from 8.2% - 12.0%  with a mean intra-class coefficient of 0.942, p < 0.001.

### Anthropometrics

Active range of motion for right/left knee extension and flexion was measured with a standard 12" goniometer to determine knee range of motion. The participant was made to lie supine with one leg extended and the other leg bent with the heel resting on table. The extended leg was measured for knee extension. Next, the measurement of the same leg was measured for flexion range of motion by having the participant raise the extended leg slightly off the table and bring the heel toward the gluteus maximus. These procedures were repeated on the opposite leg. Test to test reliability of performing these tests were 0.75-0.98. Knee circumference was measured as a general indicator of knee inflammation/swelling. The participant lied supine with one leg extended and the other leg bent with the heel resting on table. The circumference of the extended leg was measured and then repeated on the opposite leg. Measurements were performed utilizing a Gulick anthropometric tape (*Model J00305, Lafayette Instruments, Lafayette, IN*) at the joint line of both knees. Test to test reliability of performing these tests were 0.86-0.92.

### Exercise capacity

Resting heart rate was determined by palpitation of the radial artery using standard procedures [32]. Blood pressure was assessed by auscultation of the brachial artery using a mercurial sphygmomanometer using standard clinical procedures [32]. Resting heart rate and blood pressure measurements were taken on the participant in the supine position after resting for 5-min. Participants were attached to the Quinton 710 ECG (*Quinton Instruments, Bothell, WA*) and walked on a Trackmaster TMX425C treadmill (*JAS Fitness Systems, Newton, KS*). Resting expired gases were collected using the Parvo Medics 2400 TrueMax Metabolic Measurement System. The participant then performed a standard symptom-limited maximal Bruce treadmill exercise test according to standard procedures [32]. Calibration of gas and flow sensors was completed every morning prior to testing and was found to be within 3% of the previous calibration point.

A standard isotonic Olympic bench press (*Nebula Fitness, Versailles, OH*) was used for the isotonic bench press tests. A one repetition maximum (1 RM) test was performed using standard procedures. Following determination of the participants 1RM, subjects performed a bench press muscular endurance test at 70% of 1RM. Test to test reliability of performing these strength tests in our lab on resistance-trained participants have yielded low mean coefficients of variation and high reliability for the bench press (1.9%, intra-class r = 0.94).

Isokinetic testing was performed using the Biodex Multijoint Isokinetic Testing System (*Biodex Medical Systems, Shirley, NY*) to measure knee strength and endurance. Isokinetic strength was assessed bilaterally. Testing began from a dead stop with the participants' leg at 90 degrees of flexion and consisted of five, ten, and fifteen maximal voluntary concentric reciprocal knee extension and flexion repetitions at three different test speeds. Velocities were presented in a fixed order at 60, 180 and 300 degrees per second with one-minute rest between bouts. Fatigue index was calculated as the change in average force produced from the first to last third of each set of work performed. Positive values represent the percentage decline in force generation over the set while negative values represent an increase in average force generated at the latter third of the set of repetitions. Test-to-test reliability data for women with osteoarthritis has been reported to vary from 0.83 to 0.94 [33].

### Balance and functional assessment

Measurements of balance and functional capacity were obtained using the Neurocom SmartEquitest^® ^(*Neurocom International, Portland, OR*). Data were collected on postural balance and mobility utilizing the sit to stand, step up and over, and forward lunge tests following standardized procedures. Test-to-test reliability in women aged 65-75 has been reported to be r = 0.92 [34].

### Blood collection and analysis

Fasted whole blood and serum samples were collected using standard phlebotomy techniques. Whole blood samples were analyzed for complete blood counts with platelet differentials using an Abbott Cell Dyn 3500 (*Abbott Laboratories, Abbott Park, IL*) automated hematology analyzer. Serum samples were analyzed for a complete metabolic panel using a calibrated Dade Behring Dimension RXL (*Siemans AG, Munich, Germany*) automated clinical chemistry analyzer. Coefficient of variation (CV) for the tests using this analyzer was similar to previously published data for these tests (range: 1.0 to 9.6%) [35].

Serum C-Reactive Protein, IL-6, TNF-α, cortisol, and leptin were determined with either enzyme linked immuno-absorbent assay (ELISA) or enzyme immuno-absorbent assay (EIA) kits (*Cayman Chemical, AnnArbor, MI; Diagnostic Systems Laboratories, Webster, TX*) using a Wallac Victor-1420 microplate reader (*Perkin-Elmer Life Sciences, Boston, MA*) at a dual wavelength absorbance of 405 or 450 nm according to kit specifications. Intra-assay and inter-assay coefficient of variation were, respectively, 5.3-6.7% and 8.2-9.7% for TNF-α; 4.7-8.3% and 6.70-10.0% for IL-6; 6.9% and 13.1% for C-Reactive protein; and, 2.4-10.3%, and 8.0-12.0% for cortisol. The homeostasis model assessment for estimating insulin resistance (HOMA_IR_) was calculated as the product of fasting glucose times fasting insulin expressed in conventional units divided by 405 [36].

### Psychosocial and pain questionnaires

Participants completed the SF-36 Quality of Life (QOL) inventory to determine changes in quality of life scores throughout the length of the study [37]. The SF-36 QOL inventory assesses a number of physical and mental components including physical functioning (i.e., ability to perform most vigorous physical activities without limitation to health); role physical (i.e., ability to work and perform daily activities); bodily pain (i.e., limitations due to pain); general health (i.e., assessment of personal health); vitality (i.e., feelings of energy); social functioning (i.e., ability to perform normal social activities); role emotion (i.e., problems with work or other daily activities); and, mental health (state of feelings of peacefulness, happiness, and calm). This instrument has been shown to be a valid indicator of psychosocial dimensions that may be influenced by general improvements in health and/or weight loss. Perceived knee pain was determined using a Visual Analogue Scale (VAS) following procedures developed by Denegar & Perrin [38]. In addition, the Western Ontario and McMasters University Osteoarthritis Index (*WOMAC™ 3.1 Index*) was used to assess dimensions of pain, joint stiffness and disability in knee and hip osteoarthritis using a battery of 24 questions [39].

### Statistical analysis

Baseline demographic data (i.e., age, height, weight, percent body fat, BMI) were analyzed by one-way analysis of variance (ANOVA). Data were normally distributed and did not require transformation prior to statistical analysis. Related variables were grouped together and analyzed by multivariate analysis of variance (MANOVA) with repeated measures (*PASW Statistics 18.0.2 [Release April 2, 2010], SPSS Headquarters, Chicago, IL*). Non-correlated variables were analyzed by repeated measures ANOVA. Delta values were calculated and analyzed on select variables by ANOVA for repeated measures to assess changes from baseline values. Data were considered statistically significant when the probability of type I error was 0.05 or less. In some instances, quadratic interaction p-levels are reported indicating that non-linear but significant differences were observed between groups over time. Tukey's Least Significant Difference (LSD) post-hoc analyses were performed when a significant interaction was observed to determine where significance was obtained. Effect sizes were calculated using Cohen's d statistic to quantify the size and significance that may exist between groups independent of group size. Power calculations on changes observed in WOMAC scores indicated that an n-size of 8-10 per group would yield sufficient power (> 0.8) values. Additionally, power calculations on weight loss changes previously observed in similar studies indicated that a sample size of 10-15 per group yielded moderate to high power (> 0.8) values [20-22].

## Results

A total of 30 participants completed the study (54 ± 9 yrs, 163 ± 6 cm, 88.6 ± 13 kg, 46.1 ± 3% fat, 33.3 ± 5 kg/m^2^). Of these, 16 participants in the GCM group completed the study (52 ± 10 yrs, 164 ± 7 cm, 89.7 ± 13 kg, 45.9 ± 3% fat, 33.3 ± 4 kg/m^2^) while 14 participants in the P group completed the study (57 ± 7 yrs, 162 ± 6 cm, 87.3 ± 14 kg, 46.4 ± 4% fat, 33.2 ± 5 kg/m^2^). No significant differences were observed between groups on baseline demographic data.

### Energy intake

Table 1 presents dietary intake data observed for the diet and supplement groups. The diet intervention significantly reduced energy intake in both groups over time. As expected, carbohydrate intake was greater in the HC group while protein intake was greater in the HP group during the 10-week diet phase. Dietary supplementation had no influence on macronutrient intake.

**Table 1:** Dietary intake data for the diet and supplement groups

Variable	Group	0	Week 10	14	p-value
Energy Intake (kcals/d)	HC-GCM	2,356 ± 690	1,906 ± 571	2,001 ± 241	D = 0.08
	HC-P	1,760 ± 695	1,689 ± 439	1,837 ± 617	S = 0.64
	HP-GCM	1,775 ± 424	1,398 ± 411	1,441 ± 295	T = 0.06_q_
	HP-P	1,696 ± 361	1,562 ± 165	1,903 ± 274	T × D = 0.80
		
	HC	1,987 ± 730	1,768 ± 475	1,896 ± 503	T × S = 0.18
	HP	1,746 ± 377	1,459 ± 333	1,614 ± 358	T × D × S = 0.94
		
	GCM	2,046 ± 610	1,623 ± 527	1,690 ± 390	
	P	1,741 ± 593	1,651 ± 372	1,857 ± 521	
		
	Mean	1,886 ± 605	1,638 ± 439†	1,778 ± 459	
	
Carbohydrate (g/d)	HC-GCM	342 ± 103	228 ± 87	248 ± 57	D = 0.02
	HC-P	189 ± 82	218 ± 70	238 ± 117	S = 0.94
	HP-GCM	191 ± 65	125 ± 61	151 ± 38	T = 0.015 _q_
	HP-P	216 ± 39	143 ± 106	269 ± 58	T × D = 0.63
		
	HC	245 ± 115	221 ± 72	241 ± 96	T × S = 0.07
	HP	200 ± 55	132 ± 76	196 ± 84	T × D × S = 0.12_q_
		
	GCM	256 ± 11	171 ± 87†	194 ± 67	
	P	197 ± 71	196 ± 84	247 ± 100†	
	Mean	226 ± 94	184 ± 85†	222 ± 88	
	
Protein (g/d)	HC-GCM	88 ± 24	81 ± 22	75 ± 20	D = 0.22
	HC-P	76 ± 24	77 ± 16	79 ± 22	S = 0.97
	HP-GCM	79 ± 4	101 ± 31	83 ± 14	T = 0.019_q_
	HP-P	63 ± 11	133 ± 70	76 ± 11	T × D = 0.017_q_
		
	HC	80 ± 23	77 ± 16	78 ± 20	T × S = 0.35
	HP	73 ± 10	113 ± 47†	80 ± 13	T × D × S = 0.19_q_
		
	GCM	83 ± 16	92 ± 28	80 ± 16	
	P	72 ± 21	94 ± 44	78 ± 19	
		
	Mean	77 ± 19	93 ± 37†	79 ± 17	
	
Fat (g/d)	HC-GCM	78 ± 24	78 ± 24	82 ± 10	D = 0.25
	HC-P	70 ± 39	59 ± 18	62 ± 19	S = 0.26
	HP-GCM	79 ± 21	52 ± 21	59 ± 22	T = 0.085_q_
	HP-P	65 ± 32	53 ± 6	63 ± 8	T × D = 0.50
		
	HC	73 ± 33	65 ± 20	69 ± 19	T × S = 0.85
	HP	74 ± 24	53 ± 16	60 ± 18	T × D × S = 0.33
		
	GCM	79 ± 21	63 ± 23	69 ± 21	
	P	63 ± 35	60 ± 15	62 ± 16	
		
	Mean	73 ± 29	60 ± 19†	65 ± 18	

Data are means ± standard deviations. HC = high carbohydrate diet, HP = high protein diet, GCM = glucosamine/chondroitin/MSM group, P = placebo group, FFM = fat free mass, REE = resting energy expenditure, D = diet, S = supplement, T = time. q = quadratic alpha level. † Indicates p < 0.05 difference from baseline.

### Body composition and resting energy expenditure

**Figure 2:**
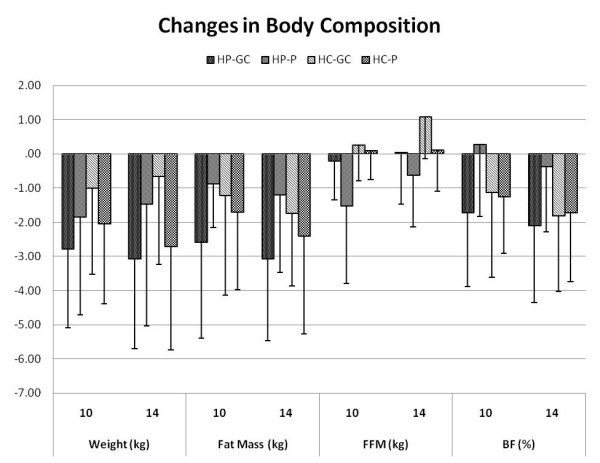
**Changes in body composition variables among groups after 10 and 14 weeks of dieting and training**.

Table 2 presents body composition and REE results observed among groups during the course of the study while Figure 2 presents changes from baseline in body composition values. Dieting and training significantly decreased body mass (-2.1 ± 3 kg or -2.4 ± 3%), fat mass (-2.3 ± 2.4 kg or -6.0 ± 6%), and percent body fat (-1.6 ± 1.9% or -3.5 ± 4%) in both groups over time while fat free mass and REE were maintained. Type of diet and supplementation had no significant effects on changes in body composition or REE.

**Table 2:** Body composition and resting energy expenditure data

Variable	Group	0	Week 10	14	p-value
Weight (kg)	HC-GCM	88.0 ± 14	87.0 ± 16	87.4 ± 13	D = 0.75
	HC-P	86.8 ± 13	84.8 ± 14	84.1 ± 13	S = 0.70
	HP-GCM	91.0 ± 13	89.2 ± 14	87.9 ± 13	T = 0.001
	HP-P	88.2 ± 17	86.4 ± 15	86.8 ± 15	T × D = 0.60
		
	HC	87.4 ± 13	85.8 ± 14	85.5 ± 14	T × S = 0.84
	HP	90.0 ± 14	87.6 ± 14	87.5 ± 13	T × D × S = 0.10
		
	GCM	89.7 ± 13	87.6 ± 14	87.7 ± 14	
	P	87.3 ± 14	85.3 ± 14	85.1 ± 13	
		
	Mean	88.6 ± 13	86.6 ± 14†	86.5 ± 13†	
	
Fat Mass (kg)	HC-GCM	37.5 ± 7	36.3 ± 9	35.8 ± 8	D = 0.81
	HC-P	37.8 ± 8	36.1 ± 9	35.4 ± 8	S = 0.98
	HP-GCM	38.9 ± 6	36.4 ± 7	35.9 ± 6	T = 0.001
	HP-P	38.0 ± 8	37.1 ± 8	36.8 ± 8	T × D = 0.93
		
	HC	37.7 ± 8	36.2 ± 8	35.6 ± 8	T × S = 0.53
	HP	38.6 ± 6	36.6 ± 7	36.2 ± 8	T × D × S = 0.19
		
	GCM	38.3 ± 6	36.3 ± 7	35.8 ± 7	
	P	37.9 ± 8	36.5 ± 8	35.9 ± 8	
	
	Mean	38.1 ± 7	36.4 ± 8†	35.9 ± 7†	
FFM (kg)	HC-GCM	44.4 ± 7	44.7 ± 8	45.5 ± 8	D = 0.74
	HC-P	42.8 ± 6	42.8 ± 7	42.8 ± 6	S = 0.45
	HP-GCM	45.7 ± 7	45.5 ± 7	45.8 ± 8	T = 0.57
	HP-P	44.5 ± 7	42.9 ± 6	43.8 ± 7	T × D = 0.09
		
	HC	43.5 ± 7	43.6 ± 7	44.0 ± 7	T × S = 0.12
	HP	45.3 ± 7	44.6 ± 6	45.1 ± 7	T × D × S = 0.77
		
	GCM	45.2 ± 7	45.1 ± 7	45.6 ± 8	
	P	43.4 ± 6	42.9 ± 6	43.2 ± 6	
		
	Mean	44.3 ± 7	44.1 ± 7	44.5 ± 7	
	
Body Fat (%)	HC-GCM	45.7 ± 3	44.6 ± 3	43.9 ± 3	D = 0.98
	HC-P	46.7 ± 4	45.5 ± 4	45.0 ± 3	S = 0.41
	HP-GCM	46.0 ± 3	44.3 ± 3	43.9 ± 3	T = 0.001
	HP-P	45.8 ± 2	46.1 ± 3	45.4 ± 2	T × D = 0.46
		
	HC	46.3 ± 4	45.1 ± 4	44.5 ± 3	T × S = 0.21
	HP	45.9 ± 2	44.9 ± 2	44.4 ± 3	T × D × S = 0.25
		
	GCM	45.9 ± 3	44.4 ± 3	43.9 ± 3	
	P	46.4 ± 4	45.7 ± 4	45.1 ± 4	
		
	Mean	46.1 ± 3	45.0 ± 3†	44.5 ± 3†	
	
REE (kcals/d)	HC-GCM	1,548 ± 262	-	1,453 ± 302	D = 0.73
	HC-P	1,400 ± 180	-	1,388 ± 218	S = 0.35
	HP-GCM	1,517 ± 301	-	1,519 ± 310	T = 0.26
	HP-P	1,477 ± 301	-	1,410 ± 147	T × D = 0.78
		
	HC	1,465 ± 225	-	1,416 ± 251	T × S = 0.93
	HP	1,504 ± 289	-	1,485 ± 268	T × D × S = 0.32
		
	GCM	1,530 ± 276	-	1,490 ± 298	
	P	1,424 ± 213	-	1,394 ± 193	
		
	Mean	1,482 ± 251	-	1,447 ± 257	

Data are means ± standard deviations. HC = high carbohydrate diet, HP = high protein diet, GCM = glucosamine/chondroitin/MSM group, P = placebo group, FFM = fat free mass, REE = resting energy expenditure, D = diet, S = supplement, T = time. † Indicates p < 0.05 difference from baseline.

### Knee anthropometric measurements

Table 3 presents knee range of motion and circumference data. No significant time × diet, time × supplement, or time × diet × supplement interactions were observed among groups in knee range of motion or circumference measures. However, left leg knee extension and flexion range of motion was significantly improved over time in both groups as a result of training.

**Table 3:** Knee range of motion data and circumference data for the diet and supplement groups

Variable	0	Weeks 10	14	Group	p-level Time	G × T
***Range of Motion***						
Extension - RL (deg)	3.02 ± 2.6	4.20 ± 3.0	4.05 ± 3.1	0.12	0.13	0.56
Extension - LL (deg)	3.02 ± 2.6	4.34 ± 3.2†	4.11 ± 3.2	0.66	0.06	0.35
Flexion - RL (deg)	123.9 ± 7	125.2 ± 7	121.6 ± 8	0.33	0.34	0.07
Flexion - LL (deg)	121.2 ± 8	126.3 ± 6†	126.7 ± 8†	0.80	0.001	0.33
***Circumference***						
Right Knee (cm)	36.9 ± 3	36.6 ± 3	37.8 ± 5	0.82	0.34	0.20
Left Knee (cm)	36.6 ± 4	36.6 ± 3	39.1 ± 5	0.92	0.06	0.18

Data are means ± standard deviations for time main effects. RL = right leg, LL = left leg, G = group, T = time. † Indicates p < 0.05 difference from baseline.

### Exercise capacity

Table 4 shows peak aerobic capacity, upper body muscular strength, and upper body muscular endurance data observed throughout the study. Exercise training significantly increased symptom-limited peak VO_2 _(5%), bench press 1RM strength (12%), and upper body bench press muscular endurance at 70% of 1RM (20%). Peak aerobic capacity was increased to a greater degree in the HP and GCM groups. No significant time × diet, time × supplement, or time × diet × supplement interactions were observed among groups in bench press 1RM strength or endurance. However, participants in the HP group produced more total lifting volume during the muscular endurance test than those in the HC group. Exercise training, diet, and supplementation had no effects on resting heart rate, systolic blood pressure or diastolic blood pressure.

**Table 4:** Exercise performance related data for the diet and supplemented groups

Variable	Group	0	Week 10	14	p-value
Peak VO_2_	HC-GCM	19.4 ± 3	19.9 ± 4	20.5 ± 3†	D = 0.85
(ml/kg/min)	HC-P	18.3 ± 5	18.5 ± 6	19.6 ± 4†	S = 0.20
	HP-GCM	20.2 ± 4	21.4 ± 4	21.9 ± 3†*	T = 0.05
	HP-P	18.7 ± 4	18.8 ± 2	16.9 ± 3†*	T × D = 0.03
		
	HC	18.8 ± 4	19.1 ± 5	20.0 ± 4†	T × S = 0.008
	HP	19.8 ± 4	20.6 ± 4†*	20.3 ± 4†	T × D × S = 0.003
		
	GCM	19.9 ± 3	20.8 ± 4†*	21.3 ± 3†*	
	P	18.4 ± 5	18.6 ± 5	18.8 ± 4	
		
	Mean	19.2 ± 4	19.8 ± 4	20.1 ± 4†	
	
Bench Press	HC-GCM	26.9 ± 5	29.1 ± 8	29.8 ± 8	D = 0.57
1RM (kg)	HC-P	27.0 ± 7	28.2 ± 6	29.5 ± 6	S = 0.19
	HP-GCM	29.8 ± 6	33.8 ± 7	34.6 ± 6	T = 0.001
	HP-P	24.4 ± 2	28.4 ± 3	27.8 ± 5	T × D = 0.18_q_
		
	HC	27.0 ± 6	28.7 ± 7	29.7 ± 7	T × S = 0.57
	HP	28.1 ± 5	32.1 ± 6	32.5 ± 6	T × D × S = 0.75
		
	GCM	28.5 ± 6	31.8 ± 7	32.5 ± 7	
	P	26.2 ± 6	28.7 ± 7	29.0 ± 6	
		
	Mean	27.5 ± 6	30.2 ± 6†	30.9 ± 7†	
	
Upper Body Endurance (kg)	HC-GCM	206 ± 52	269 ± 121	245 ± 120	D = 0.81
	HC-P	164 ± 88	175 ± 109	198 ± 142	S = 0.02
	HP-GCM	242 ± 81	299 ± 128	278 ± 116	T = 0.04_q_
	HP-P	157 ± 22	179 ± 34	153 ± 26	T × D = 0.59
		
	HC	182 ± 75	216 ± 120	219 ± 131	T × S = 0.17_q_
	HP	216 ± 66	262 ± 120	240 ± 113	T × D × S = 0.64
		
	GCM	226 ± 59	286 ± 122	264 ± 115	
	P	162 ± 73	176 ± 90	184 ± 119	
		
	Mean	197 ± 72	237 ± 120†	228 ± 121	

Data are means ± standard deviations. HC = high carbohydrate, HP = high protein, GCM = glucosamine/chondroitin/MSM, P = placebo, HR = heart rate, SBP = systolic blood pressure, DBP = diastolic blood pressure, VO_2 _**= **oxygen uptake, 1 RM = one repetition maximum, D = diet, S = supplement, T = time, q = quadratic alpha level. † Indicates p < 0.05 difference from baseline. * represents p < 0.05 difference between groups.

Results from isokinetic knee extension and flexion tests are presented in Table 5. No significant group or group × time interactions were observed. Therefore, data are presented for mean time effects. Training significantly increased knee extension and flexion peak torque values in each set of maximal voluntary contractions studied. Average gains in knee extension peak torque strength was 8-13% when performing 5 repetitions at 60 deg/sec, 12-22% when performing 10 repetitions at 180 deg/sec, and 12-19% when performing 15 repetitions at 300 deg/sec. Similarly, knee flexion peak torque increased by 26-28%, 45-46%, and 30-38% during the three exercise bouts, respectively. There was also evidence that training influenced fatigue index responses.

**Table 5:** Mean isokinetic knee extension and flexion data observed over time

Variable	0	Weeks 10	14	Group	p-level Time	G × T
***5 Repetitions at 60 deg/sec***						
Peak Torque - RL Extension (kg/m)	9.90 ± 2.0	10.38 ± 2.6	10.69 ± 2.8	0.36	0.13	0.69
Peak Torque - LL Extension (kg/m)	9.15 ± 2.2	10.38 ± 2.6†	10.34 ± 2.9†	0.47	0.04	0.44
Peak Torque - RL Flexion (kg/m)	4.66 ± 1.6	5.53 ± 1.6†	5.99 ± 2.1†	0.62	0.003	0.90
Peak Torque - LL Flexion (kg/m)	4.44 ± 1.6	5.47 ± 1.7†	5.61 ± 1.9†	0.71	0.01	0. 45
Fatigue Index - RL Extension (%)	-0.8 ± 50	9.4 ± 18	8.7 ± 25	0.79	0.32	0.54
Fatigue Index - LL Extension (%)	3.5 ± 30	11.1 ± 19	11.0 ± 18	0.73	0.38	0.41
Fatigue Index - RL Flexion (%)	-8.8 ± 72	16.9 ± 28†	25.3 ± 13†	0.23	0.02	0.28
Fatigue Index - LL Flexion (%)	12.6 ± 30	19.4 ± 18	23.4 ± 10	0.82	0.12	0.75
***10 Repetitions at 180 deg/sec***						
Peak Torque - RL Extension (kg/m)	6.05 ± 2.3	6.45 ± 2.4	6.82 ± 2.4†	0.12	0.05	0.40
Peak Torque - LL Extension (kg/m)	5.60 ± 2.8	6.40 ± 2.7	6.85 ± 2.3†	0.47	0.04	0.44
Peak Torque - RL Flexion (kg/m)	2.80 ± 1.5	3.70 ± 1.8†	4.10 ± 1.9†	0.35	0.001	0.66
Peak Torque - LL Flexion (kg/m)	2.68 ± 1.7	3.49 ± 1.6†	3.90 ± 1.7†	0.60	0.001	0. 48
Fatigue Index - RL Extension (%)	-1.9 ± 33	-9.6 ± 67	9.5 ± 26	0.19	0.12	0.84
Fatigue Index - LL Extension (%)	-17.6 ± 55	5.2 ± 27†	-0.2 ± 47†	0.08	0.02	0.49
Fatigue Index - RL Flexion (%)	-12.1 ± 84	7.9 ± 56†	17.7 ± 22†	0.37	0.08	0.53
Fatigue Index - LL Flexion (%)	-48.9 ± 139	9.8 ± 53†	9.7 ± 67†	0.61	0.02	0.44
***15 Repetitions at 300 deg/sec***						
Peak Torque - RL Extension (kg/m)	32.6 ± 13	36.6 ± 14	36.2 ± 15	0.68	0.17	0.39
Peak Torque - LL Extension (kg/m)	31.0 ± 16	36.2 ± 15†	37.0 ± 15†	0.62	0.02	0.12
Peak Torque - RL Flexion (kg/m)	14.8 ± 11	19.0 ± 13†	19.3 ± 13†	0.76	0.02	0.61
Peak Torque - LL Flexion (kg/m)	12.7 ± 11	17.2 ± 12†	17.6 ± 11†	0.82	0.02	0. 24
Fatigue Index - RL Extension (%)	7.8 ± 43	10.8 ± 27	17.2 ± 29	0.46	0.19	0.83
Fatigue Index - LL Extension (%)	4.0 ± 48	11.3 ± 24	17.6 ± 37	0.46	0.25	0.77
Fatigue Index - RL Flexion (%)	-2.0 ± 94	14.1 ± 70	17.9 ± 68†	0.52	0.36	0.82
Fatigue Index - LL Flexion (%)	-20.2 ± 103	16.3 ± 89†	19.1 ± 62†	0.76	0.02	0.94

Data are means ± standard deviations for time main effects. RL = right leg, LL = left leg, G = group, T = time. † Indicates p < 0.05 difference from baseline.

### Balance and functional capacity

Table 6 presents functional balance testing results. No significant group or group × time interactions were observed. Therefore, data are presented for mean time effects. Training had no effects on weight transfer, rising index, or sway velocity measures obtained during the sit to stand test. However, lift-up index increased by 8-12% and movement time decreased by 15% in the step up and over knee function test. In the forward lunge knee function test, lunge distance was significantly increased (7-9%) while contact time (-19 to -20%) and force impulse (-17 to -19%) values decreased.

**Table 6:** Functional balance testing results observed over time

Variable	0	Weeks 10	14	Group	p-level Time	G × T
***Sit to Stand Function***						
Weight Transfer (sec)	0.377 ± 0.18	0.355 ± 0.17	0.370 ± 0.22	0.80	0.91	0.89
Rising Index (% body weight)	16.6 ± 4.3	18.6 ± 5.7	18.2 ± 5.6	0.97	0.13	0.34
Sway Velocity (deg/sec)	4.63 ± 1.3	4.56 ± 1.1	4.62 ± 1.2	0.78	0.78	0.12
***Step Up and Over Knee Function***						
Lift-up Index - RL (% body weight)	41.2 ± 9.2	43.6 ± 9.7	44.5 ± 8.6†	0.90	0.01	0.71
Lift-up Index - LL (% body weight)	34.7 ± 8.5	37.4 ± 8.1	38.9 ± 7.2†	0.70	0.002	0.50
Impact Index - RL (% body weight)	48.7 ± 11.2	48.4 ± 12.1	48.3 ± 10.9	0.91	0.70	0.77
Impact Index - LL (% body weight)	52.1 ± 10.6	52.4 ± 13.5	54.5 ± 14.1	0.84	0.22	0.47
Movement Time - RL (sec)	1.73 ± 0.3	1.55 ± 0.2†	1.47 ± 0.2†	0.83	0.001	0.07
Movement Time - LL (sec)	1.76 ± 0.3	1.60 ± 0.5†	1.49 ± 0.3†	0.98	0.002	0.68
***Forward Lunge Knee Function***						
Distance - RL (% body height)	43.2 ± 7	45.6 ± 7†	46.3 ± 7†	0.14	0.001	0.75
Distance - LL (% body height)	42.5 ± 5	45.7 ± 7†	46.2 ± 7†	0.24	0.001	0.49
Impact Index - RL (% body weight)	18.5 ± 5	19.0 ± 7	18.6 ± 5	0.65	0.76	0.77
Impact Index - LL (% body weight)	14.0 ± 5	16.4 ± 7	15.7 ± 5	0.86	0.15	0.97
Contact Time - RL (sec)	1.664 ± 0.5	1.344 ± 0.3†	1.359 ± 0.4†	0.57	0.001	0.81
Contact Time - LL (sec)	1.641 ± 0.7	1.347 ± 0.4†	1.310 ± 0.4†	0.64	0.002	0.98
Force Impulse - RL (% body weight/sec)	168 ± 53	139 ± 36†	141 ± 38†	0.61	0.001	0. 59
Force Impulse - LL (% body weight/sec)	162 ± 65	136 ± 33†	132 ± 37†	0.62	0.002	0. 99

Data are means ± standard deviations for time main effects. RL = right leg, LL = left leg, G = group, T = time. † Indicates p < 0.05 difference from baseline.

### Blood samples

Table 7 shows serum blood and hormone markers observed among groups during the study. No significant group or group × time interactions were observed among groups. Therefore, data are presented for mean time effects. Training and dieting significantly decreased total cholesterol (-8%), low-density lipoproteins (-12%), high density lipoproteins (-12%), blood urea nitrogen (-14%), creatinine (-15%), uric acid (-9%), alanine aminotransaminase (-23%), HOMA_IR _(-17%), and leptin (-30%) values while glucose (-7%) values tended to be lower. As expected, training moderately increased creatine kinase levels (49%) and tended to increase C-reactive protein values (44%). No significant differences were observed remaining whole blood and serum markers assayed.

**Table 7:** Fasting serum blood and hormone markers observed over time

Variable	0	Weeks 10	14	Group	p-level Time	G × T
***Blood Lipids & Glucose***						
Triglycerides (mmol/l)	1.71 ± 1.0	1.59 ± 1.0	1.62 ± 1.0	0.91	0.51	0.83
Cholesterol (mmol/l)	5.61 ± 1.0	5.15 ± 0.8†	5.25 ± 1.2	0.05	0.08_q_	0.78
LDL (mmol/l)	3.65 ± 0.8	3.23 ± 0.6†	3.34 ± 0.9	0.13	0.04 _q_	0.51
HDL (mmol/l)	1.39 ± 0.3	1.23 ± 0.2†	1.24 ± 0.3†	0.14	0.02	0.96
Glucose (mmol/l)	5.93 ± 0.8	5.69 ± 0.8	5.52 ± 0.9	0.99	0.08	0.96
***Serum Protein and Enzymes***						
BUN (mmol/l)	5.09 ± 1.4	4.85 ± 1.4	4.36 ± 1.4†	0.91	0.006	0.44
Creatinine (1/2 mol/l)	72 ± 15	69 ± 13	61 ± 15†	0.66	0.003	0.68
BUN/Creatinine Ratio	17.6 ± 3.8	17.6 ± 3.7	18.0 ± 4.4	0.63	0.55	0.33
Uric Acid (1/2 mol/l)	328 ± 92	300 ± 68†	300 ± 77†	0.49	0.09	0.93
CK (IU/l)	59 ± 36	87 ± 42†	88 ± 27†	0.23	0.001	0.86
ALT (IU/l)	25.5 ± 11	19.7 ± 6†	22.0 ± 10	0.81	0.008_q_	0.14
AST (IU/l)	20.0 ± 6	20.0 ± 5	21.8 ± 8	0.95	0.17	0.96
GGT (IU/l)	42.8 ± 30	41.7 ± 32	50.9 ± 45	0.66	0.15	0.23
***Hormones***						
C-Reactive Protein (1/2 mol/l)	4.93 ± 4.3	5.12 ± 4.2	7.12 ± 6.7†	0.84	0.06	0.55
IL-6 (pg/ml)	3.68 ± 3.9	3.54 ± 4.1	3.38 ± 5.0	0.13	0.78	0.16
TNF-α (pg/ml)	0.72 ± 2.9	0.90 ± 3.5	0.96 ± 3.3	0.19	0.71	0.60
Cortisol (nmol/l)	825 ± 827	807 ± 599	846 ± 943	0.75	0.56	0.07
Insulin (pmol/l)	90.7 ± 90	96 ± 104	88 ± 98	0.13	0.58	0.81
Glucose/Insulin Ratio	18.3 ± 20	20.2 ± 26	24.1 ± 29	0.36	0.38	0.67
HOMA_IR_	3.26 ± 3.5	3.33 ± 4.0	2.69 ± 3.0†	0.07	0.05	0.42
Leptin (1/2 g/l)	185 ± 134	130 ± 86†	134 ± 93†	0.97	0.03	0.51

Data are means ± standard deviations for time main effects. LDL = low density lipoproteins, HDL = high density lipoproteins, BUN = blood urea nitrogen, CK = creatine kinase, ALT = alanine aminotransferase, AST = aspartate aminotranferase, GGT = gamma glutamyltransferase, IL-6 = interleukin 6, TNF-α = tumor necrosis factor alpha, HOMA_IR _= homeostatic model assessment of insulin resistance, G = group, T = time, q = quadratic alpha level. † Indicates p < 0.05 difference from baseline.

### Psychosocial and pain questionnaires

Table 8 presents WOMAC, VAS, and QOL results observed. No significant group or group × time interactions were observed among groups. Therefore, data are presented for mean time effects. Participants experienced significant reductions in WOMAC perceptions of pain (-53%), joint stiffness (-44%), and limitations in physical function (-49%) during the course of the study with no group or group × time interactions observed. Likewise, VAS pain was decreased by 59% during the course of the study. Trends were observed in time by diet (p = 0.10) and time × supplement (p = 0.08) interactions with a moderate to large effect size observed (d = 1.1) but results were too inconsistent to support claims that GCM supplementation lessens perceptions of knee pain in active individuals. Participants also experienced significant improvements in QOL measures of physical functioning (59%), vitality (120%), and social function (66%) with no significant differences observed among diet and supplement groups.

**Table 8:** WOMAC, VAS pain, and quality of life measures observed over time

Variable	0	Weeks 10	14	Group	p-level Time	G × T
***WOMAC***						
Pain	156 ± 81	84 ± 64†	74 ± 58†	0.81	0.001	0.46
Stiffness	84 ± 41	47 ± 44†	50 ± 40†	0.45	0.001	0.63
Physical Function	879 ± 428	517 ± 390†	449 ± 335†	0.81	0.001	0.61
***VAS***						
Pain	3.97 ± 1.9	2.51 ± 1.8†	1.78 ± 1.8†	0.18	0.001	0.43
						
***Quality of Life***						
						
Physical Function	44.4 ± 38	55.4 ± 36	70.4 ± 17†	0.47	0.004	0.93
General Health	13.3 ± 15	15.2 ± 10	16.7 ± 7	0.73	0.12	0.47
Vitality	8.3 ± 12	15.0 ± 10	18.3 ± 7†	0.06	0.001	0.88
Social Function	18.3 ± 20	26.4 ± 14	30.3 ± 9†	0.21	0.004	0.13
Mental Health	11.7 ± 4	13.5 ± 2	9.6 ± 5	0.91	0.001_q_	0.51

Data are means ± standard deviations for main time effects. WOMAC = Western Ontario and McMasters University Osteoarthritis Index, VAS = Visual Analogue Scale. † Indicates p < 0.05 difference from baseline.

## Discussion

Osteoarthritis is a degenerative disease that is characterized by focal erosive lesions, cartilage destruction, subchondral sclerosis, cyst formation, and large osteophyte formation at joint margins that result in the structural and functional failure of synovial joints [13,40]. It is the most prevalent musculoskeletal disorder diagnosed in the United States which affects nearly 15% of Americans and costs an estimated $80 billion dollars annually [41]. Athletes with prior knee injuries and individuals who maintain an active lifestyle as they age are also at risk to experience knee pain or degenerative joint issues [5,27,28]. Although the etiology of OA involves multiple factors, obesity has been identified as a primary risk factor involved in the development of the disease [9]. Individuals with a BMI greater than 30 kg/m^2 ^are four times as likely to have knee OA than those with a BMI less than 25.0 kg/m^2 ^[9]. Although the specific amount of weight loss needed to improve or prevent OA has yet to be determined, empirical research has found that for every one pound of weight loss, there is a four pound reduction in knee joint load per step [42]. With such a drastic reduction in pressure on OA affected knees, alleviating obesity through weight loss has been suggested to be among the most beneficial methods of relieving pressure on osteoarthritic joints.

Participation in a therapeutic exercise program has been reported to aid in the management of OA symptoms [12,43,44]. The American College of Sports Medicine recommends that OA patients engaged in daily static stretching exercises to improve flexibility; low intensity resistance training involving major muscle groups (10-12 repetitions, 40-60% of 1RM, 2-3 d/week); and, aerobic exercise (40-60% of peak VO_2_, up to 30-min, 3-5 d/week) as tolerated [45,46]. Regular exercise has also been reported to improve the balance and functionality of overweight and obese individuals with knee OA [8]. Therefore, exercise and weight loss have been recommended as effective strategies in managing symptoms of OA [8-10,12,13,42,43,47].

A number of studies support these recommendations. For example, Felson and colleagues [7] reported that weight loss reduced the risk for development of OA in women. Christensen and associates [10] reported that OA patients following a low-energy diet (~840 kcal/d) that included weekly dietary counseling sessions was more effective in promoting weight loss (11.1% vs. 4.3%) and improving WOMAC index scores (-35% vs. -14%) than patients educated about weight loss who maintained a moderately hypo-energetic diet (~1,200 kcal/d). Similarly, Miller and coworkers [9] reported that older obese adults with symptomatic knee OA who followed an intensive weight loss program for 6-months that included meal replacement bars and drinks (~1,000 kcal/d) experienced greater weight loss (0.1% vs. 8.5%), fat loss (0.08% vs. 23.2%); and, improvement in WOMAC scores (-5% vs. -33%), 6-min walking distance (2.3% vs. 16.7%), and stair climb time (7.5% vs. -16.3%) than those who maintained weight. Penninx and associates [47] reported that aerobic and resistance exercise may reduce and/or prevent the incidence of disability in activities of daily living in patients with knee OA. Finally, Messier and coworkers [8] examined the effects of long-term weight loss and exercise on self-reported physical function in older obese adults with knee OA. Participants followed a diet program, an exercise program that involved aerobic and resistance-exercise, a diet plus exercise intervention, or usual care. The researchers found that participants following the diet plus exercise program experienced significant improvements in self-reported physical function, 6-min walk distance, stair climb time, and knee pain compared to those in the usual care group. Exercise alone improved 6-min walk distance while dieting alone did not result in greater functional improvement than usual care.

Present findings support prior reports indicating that weight loss and exercise training provided therapeutic benefit for women with knee OA. In this regard, the circuit style resistance-training program and weight loss program used in this study promoted significant reductions in body mass (-2.4%), fat mass (-6%), and body fat (-3.5%) while increasing symptom-limited peak VO_2 _(5%), upper body 1RM strength (12%), upper body muscular endurance (20%), isokinetic knee extension and flexion peak torque (12-46%), step up and over knee function (8-15%), and forward lunge knee function (7-20%). These changes were accompanied by significant improvements in total cholesterol (-8%), low-density lipoproteins (-12%), HOMA_IR _(-17%), and leptin (-30%) values. Interestingly, reductions in serum leptin levels have been reported to be associated with improved physical function in patients with OA [48]. Participants also reported less perceptions of pain (-53%), joint stiffness (-44%), and limitations in physical function (-49%) on the WOMAC index as well as a 59% reduction in VAS pain ratings. These findings provide additional evidence that patients with knee OA may experience significant improvements in markers of health, fitness, functional capacity, and perceptions of pain when following a weight loss and exercise program that includes resistance-training.

However, present findings add to our understanding of how different types of diets and concomitant dietary supplementation with a GCM affect weight loss, training adaptations, functional capacity, and/or perceptions of pain in women with knee OA. In this regard, a number of studies have indicated that replacing carbohydrate with protein while following a hypo-energetic diet promotes greater fat loss [14,15,19,49]. The rationale has been that there are thermogenic advantages in metabolizing protein compared to carbohydrate and that a higher amount of protein in the diet can help maintain fat free mass during weight loss thereby helping minimize reductions in resting energy expenditure that is often associated with weight loss [14,16]. Our previous research examining the efficacy of the exercise and diet program used in this study provides some support to this theory [20,21,23]. Therefore, we hypothesized that women with knee OA may experience greater weight loss and therapeutic benefits from following a higher protein diet. Present findings, however, indicate that women with knee OA benefited from both a higher carbohydrate and higher protein diet. Although there was some evidence that women following the HP diet experienced greater gains in symptom-limited peak aerobic capacity, no significant differences were observed in amount of weight loss, fat loss, or resting energy expenditure when diets were compared. Participants in both groups effectively maintained fat free mass and resting energy expenditure levels despite experiencing significant reductions in weight and fat mass. Additionally, no significant differences were observed between diet types among changes in strength, muscular endurance, functional tests, or markers of health. These findings indicate that the type of diet does not appear to influence weight loss or training adaptations in sedentary obese women with knee OA initiating a weight loss and exercise training program. The lack of statistical significance could be due to the small sample-size studied and/or that the exercise stimulus was effective enough to negate any additional metabolic benefits from adherence to a higher protein diet in this population. Nevertheless, present findings do not support our hypothesis that women with knee OA may experience greater benefits from following a higher protein hypo-energetic diet.

Several studies have also indicated that glucosamine and/or chondroitin supplementation may provide therapeutic benefits in individuals with knee OA. For example, Reginster and associates [50] reported that 3-years of glucosamine sulphate supplementation (1,500 mg/d) prevented progression of joint-space narrowing and improved WOMAC scores in patients with knee OA. Similarly, Pavelka and colleagues [25] found that dietary supplementation of glucosamine sulfate (1,500 mg/d for 3-years) retarded the clinical progression of knee OA. Braham et al [51] found that 2,000 mg/d of glucosamine supplementation for 12-weeks improved markers of quality of life and self-reported perceptions of knee pain in individuals with regular knee pain. Usha and coworkers [26] reported that dietary supplementation of 1,500 mg/d of glucosamine and/or MSM for 12-weeks produced an analgesic and anti-inflammatory effect, reduced perceptions of pain, and improved functional ability of joints in patients with mild to moderate knee OA. Moreover, Matsuno and colleagues [52] investigated the effects of 12-weeks of ingesting a dietary supplement containing glucosamine hydrochloride (1,200 mg/d), shark cartiliage powder (300 mg/d), chondroitin (75-111 mg/d), and quercetin (45 mg/d) on synovial fluid properties of patients with OA. The researchers reported that the OA patients experienced significant improvements in pain symptoms, ability to perform daily activities (walking and climbing up and down stairs), and changes in synovial fluid properties. Finally, Ng and coworkers [53] reported that dietary supplementation of glucosamine sulphate (1,500 mg/d) for 6-weeks reduced OA symptoms in individuals walking a minimum of approximately 30-min per day. These findings provide support to the theory that glucosamine and chondroitin supplementation may provide some therapeutic benefits to patients with knee OA.

In the present study, subjects ingested in a double blind and randomized manner a placebo or a dietary supplement containing 1,500 mg/d of glucosamine, 1,200 mg/d of chondroitin sulfate, and 900 mg/d of MSM. We found that symptom-limited peak aerobic capacity was increased to a greater degree in participants ingesting the GCM supplement with the greatest effects observed in the HP-GCM group. In addition, mean group upper extremity muscular endurance was greater in the GCM group compared to the P group. However, GCM supplementation did not significantly affect remaining markers of isotonic or isokinetic strength, balance, functional capacity, markers of health, self-reported perceptions of pain, or indicators of quality of life. These findings indicate that GCM supplementation provides only marginal additive benefit to a resistance-based exercise and weight loss program. The lack of additive benefits observed could be due to limitations in sample size, length of the intervention, and/or the fact that the exercise intervention resulted in marked improvement in functional capacity and perceptions of pain thereby minimizing the impact of dietary supplementation of GCM. However, additional research is needed to examine the influence of GCM supplementation during a training and weight loss program before definitive conclusions can be drawn.

## Conclusions

Present findings indicate that adherence to a resistance-based circuit training and weight loss program promoted weight and fat loss, increased strength and functional capacity, and improved markers of health in sedentary obese women with clinically-diagnosed knee osteoarthritis. These findings support contentions that exercise and weight loss may have therapeutic benefits for women with knee osteoarthritis. Although some trends were observed, the type of diet and dietary supplementation of GCM provided marginal additive benefits. However, since diet and GCM supplementation appeared to affect symptom-limited peak aerobic capacity and some moderate to large effect sizes were noted in key variables, additional research with a larger sample size is needed to determine whether type of diet and/or GCM supplementation while participating in an exercise and weight loss program may provide therapeutic benefits in this population.

## Competing interests

Curves International (*Waco, TX, USA*) provided funding for this project through an unrestricted research grant to Baylor University when the Principal Investigator and the Exercise & Sport Nutrition Lab were affiliated with that institution and currently provides funding to Texas A&M University to conduct exercise and nutrition related research. All researchers involved independently collected, analyzed, and interpreted the results from this study and have no financial interests concerning the outcome of this investigation. Data from this study have been presented at the Federation of American Societies of Experimental Biology annual meeting. Publication of these findings should not be viewed as endorsement by the investigators or their institutions of the programs or materials investigated.

## Authors' contributions

TMC served as the study supervisor, oversaw all testing, and assisted in writing of the manuscript. CW assisted in data collection and manuscript preparation. CR, MF, LG, BC, CMK, KD, RL, EN, MI and MC assisted in data collection, data analysis, and/or manuscript preparation. DW oversaw analysis of blood work. LS provided input on study design and results. RBK served as Principal Investigator and contributed to the design of the study, statistical analysis, manuscript preparation, and procurement of external funding. All authors read and approved the final manuscript.
